# Social Media Surveillance of Multiple Sclerosis Medications Used During Pregnancy and Breastfeeding: Content Analysis

**DOI:** 10.2196/13003

**Published:** 2019-08-07

**Authors:** Bita Rezaallah, David John Lewis, Carrie Pierce, Hans-Florian Zeilhofer, Britt-Isabelle Berg

**Affiliations:** 1 Department of Clinical Research University of Basel Basel Switzerland; 2 Patient Safety Novartis Pharma AG Basel Switzerland; 3 School of Health and Human Sciences University of Hertfordshire Hatfield United Kingdom; 4 Booz Allen Hamilton Inc Boston, MA United States; 5 Department of Cranio-Maxillofacial Surgery University Hospital of Basel Basel Switzerland; 6 Hightech Research Center of Cranio-Maxillofacial Surgery University of Basel Basel Switzerland

**Keywords:** pharmacovigilance, machine learning, pregnancy outcome, postpartum, central nervous system agents, risk assessment, text mining

## Abstract

**Background:**

Multiple sclerosis (MS) is a chronic neurological disease occurring mostly in women of childbearing age. Pregnant women with MS are usually excluded from clinical trials; as users of the internet, however, they are actively engaged in threads and forums on social media. Social media provides the potential to explore real-world patient experiences and concerns about the use of medicinal products during pregnancy and breastfeeding.

**Objective:**

This study aimed to analyze the content of posts concerning pregnancy and use of medicines in online forums; thus, the study aimed to gain a thorough understanding of patients’ experiences with MS medication.

**Methods:**

Using the names of medicinal products as search terms, we collected posts from 21 publicly available pregnancy forums, which were accessed between March 2015 and March 2018. After the identification of relevant posts, we analyzed the content of each post using a content analysis technique and categorized the main topics that users discussed most frequently.

**Results:**

We identified 6 main topics in 70 social media posts. These topics were as follows: (1) expressing personal experiences with MS medication use during the reproductive period (55/70, 80%), (2) seeking and sharing advice about the use of medicines (52/70, 74%), (3) progression of MS during and after pregnancy (35/70, 50%), (4) discussing concerns about MS medications during the reproductive period (35/70, 50%), (5) querying the possibility of breastfeeding while taking MS medications (30/70, 42%), and (6) commenting on communications with physicians (26/70, 37%).

**Conclusions:**

Overall, many pregnant women or women considering pregnancy shared profound uncertainties and specific concerns about taking medicines during the reproductive period. There is a significant need to provide advice and guidance to MS patients concerning the use of medicines in pregnancy and postpartum as well as during breastfeeding. Advice must be tailored to the circumstances of each patient and, of course, to the individual medicine. Information must be provided by a trusted source with relevant expertise and made publicly available.

## Introduction

### Background

Multiple sclerosis (MS) is a chronic disease of the central nervous system [1]. It is more prevalent in females than males, with a ratio of approximately 3:1 [2]. Female MS patients are predominantly of childbearing potential with the average age of disease onset being 29.2 years [1]. The prevalence of MS is more common further from the equator; this maybe because of vitamin D deficiency rather than only genetics [3]. Pregnancy is not contraindicated in MS but remains a concern among female patients for a variety of reasons [2]. Pregnancy appears to have a protective effect in MS such that pregnant women suffer a reduced number of MS relapses, especially during the third trimester (reduction of around 70%). Thereafter, relapse rates tend to increase in the first 3 months postpartum [4,5]. However, this protective effect of pregnancy and the risk of postpartum relapse are both related to each patient’s MS history and current disease activity [6].

Pregnant women are usually excluded from clinical trials because of ethical issues [7]; thus, safety information about human drug exposure during pregnancy is very limited at the time a marketing authorization is granted [8]. Pregnancy registries have been developed to address this gap in the safety profile of newly authorized medicines. Despite the evident advantages, such registries often suffer from low enrollment, resulting in delayed findings, selection bias, heterogeneity in data collection methods, and high costs [9]. As a result, prescribing information and patient information leaflets contain limited safety information for pregnant and breastfeeding patients [8]. Despite the evident need, to the best of our knowledge, there are no globally accepted guidelines by regulatory agencies for the medical management of MS during pregnancy and breastfeeding.

The rapid expansion of the internet and the availability of various social media platforms in recent years has increased the frequency with which patients use the internet [10]. In the United States, 90% of adults use the internet regularly, and 72% have searched for health information online [11]. Pregnant women in particular often access the internet to seek health information [12]. A cohort of pregnant women has been identified on Twitter using text mining and machine learning [13]. The availability of data for this cohort of pregnant women in social media provides an opportunity to explore and gain further insights into patient experiences related to MS medications. Therefore, by increasing health care professionals’ (HCPs’) awareness of patient concerns, carers can better advise patients during clinical visits.

### Objective

The objective of this study was to analyze data qualitatively and describe the content of posts in online pregnancy forums to understand better patient experiences resulting from the use of MS medications during pregnancy, postpartum, and breastfeeding.

## Methods

### Data Acquisition and Classification

We obtained data from publicly available online pregnancy forums. An existing digital monitoring platform called MedWatcher Social (now called Epidemico, Booz Allen Hamilton) was utilized; this system has been described elsewhere [10,13,14]. MedWatcher Social comprises the natural language processing component that acquires public data from the internet, applies classification algorithms, and extracts adverse event–related posts. The aggregated frequency of product-event pairs identified by MedWatcher was concordant with data from the public US Food and Drug Administration (FDA) Adverse Event Reporting System by System Organ Class [9].

The classifier was designed to automatically collect, classify, and analyze social media discussions and threads pertaining to medicinal products [10,13,14]. The system collected online forum posts both retrospectively and prospectively via authorized third-party data vendors using the names of medicinal products as search terms. After data ingestion, a naïve Bayes classifier scored and filtered each post according to its relevance. Using statistical machine learning and a training set of over 360,000 hand-labeled social media posts, the classifier was trained to recognize the following:

- Descriptions of adverse drug reactions
- Medication errors
- Product quality issues
- Other patient experiences with medicinal products

The classifier was also used to exclude “noise” (eg, nonvalid product posts and spam). After filtering the data, natural language processors were applied to recognize and extract product and symptom terms through tokenization and proprietary taxonomies. References to products were standardized and consolidated, and vernacular descriptions of medical concepts were translated into the best matched term within the Medical Dictionary for Regulatory Activities terminology [15]. For this analysis, we identified and extracted a dataset from the system comprising posts acquired from 21 publicly accessible pregnancy social media forums listed in Textbox 1, published between March 2015 and March 2018. The forum data were acquired using third-party data from vendors, namely, Socialgist (SocialGist) and Datasift (DataSift Inc), and thus, was dependent on availability from those vendors. Data were not randomly sampled; rather, we selected any forums that were both available from Socialgist or Datasift and were dedicated to discussions around pregnancy or breastfeeding. The classifier used for the analysis was trained only on English language data, so we only used English language posts for this analysis.

In addition, we identified a list of products authorized for the treatment of MS and filtered the data accordingly. The products were alemtuzumab, teriflunomide, interferon beta-1a, interferon beta-1b, glatiramer acetate, daclizumab, dimethyl fumarate, fingolimod, and natalizumab. Posts mentioning either the active substance or brand name of each medicinal product were collected as shown in Multimedia Appendix 1.

**List of publicly available pregnancy forums.**

- Babiesbase.com
- Babycenter.com
- Babycenter.com.au
- Babycentre.co.uk
- Cafemom.com
- Dcurbanmom.com
- Fertility.org
- Magrossesse.com
- Mumsnet.com
- Whattoexpect.com
- Swissmomforum.ch
- Baby-cafe.cz
- Babycenter.ca
- Babycenter.in
- Circleofmoms.com
- Essentialbaby.com.au
- Justmommies.com
- Netmums.com
- Thebump.com
- Fertilethoughts.com
- Scarymommy.com

### Content Analysis

After automated classification, reports were manually divided into 2 groups: discussions related to pregnancy or breastfeeding and posts containing no thread relevant to pregnancy or lactation. In this study, we focused only on posts where an individual wrote about an experience related to a current or previous pregnancy, breastfeeding related to medicinal treatment of MS medication, a complication of MS or treatment of this disease.

A human expert reviewed the posts to characterize the experiences described in each post. First, we collected any medical information that a user shared in a post, such as time since their diagnosis of MS, planned or unplanned pregnancy, gestational age, outcome of pregnancy (or multiple pregnancies), number of pregnancies, current or previous pregnancy, concomitant medications, and John Cunningham (JC) virus serology results. Second, for the questions and concerns written in posts, we applied the content analysis method [16,17]. The aim was to use this categorization to identify common themes (threads) and to assess their frequency. To start with, we used open coding for obtaining the sense of the content. The coding team was composed of a physician (BR), a pharmacovigilance expert (DL), a statistician (AZ), and a machine-learning expert (CP). We created a codebook based on features that individual users shared (eg, what were their concerns and what action was taken with the medications). Subsequently the initial codes formed higher order headings of main topics. The entire dataset was reviewed, and posts were assigned to each topic. In addition, we quantified the content by measuring the frequency of each topic, which we cautiously proposed may stand as a proxy for significance [17].

The unit of analysis was the number of posts. It should be noted that in each individual post, the author might have provided comments on more than 1 main topic.

### Ethics Statement

All human subject data used in this analysis were publicly available and have been presented in a deidentified format; in no case was any personally identifiable information (PII) reviewed. In fact, the classifier was set up to deidentify individual posts by removing any text relating to PII. We did not contact any individual on social media for follow-up as we felt that this posed unacceptable ethical and potential data privacy concerns. Thus, all of the posts were evaluated without knowledge of the identity of the patients involved.

## Results

### Data Processing Results

Our initial dataset comprised 376,691 posts that had been shared publicly on the pregnancy forums during the 4-year period of observation. This dataset was reduced to 168 (0.04% of total) posts relevant to pregnancy or breastfeeding and MS after filtering for posts mentioning the specified products. Finally, 16 posts containing spam-like language, non-English text, and nonvalid mentions of the product were automatically identified as irrelevant and were filtered out, leaving 152 posts for analysis as shown in Table 1.

Among the 152 posts, 70 unique posts discussed a current or previous pregnancy and breastfeeding experiences related to MS medications. The remaining 82 posts were noninformative concerning pregnancy and breastfeeding. As a result, we focused on the 70 posts that provided pertinent and substantive information. Table 2 provides illustrative examples of medically relevant information shared by the post authors. We could not identify the gender of individual users in each post but based on the content and the way that the text related personal sentiments and explanations, we assumed that it was predominantly pregnant women who authored the content.

Patients indicated that their newborn children were healthy, with no reports of congenital anomalies, in 18 of 70 posts (25%). MS patients shared in 22 of 70 (31%) posts their gestational age, and in 21 of 70 (30%) posts the year of the first diagnosis of MS was mentioned.

**Table 1:** Result of data processing.

Number of posts extracted from database via automation	Before spam removal, n	After spam removal, n
Posts mentioning any product	376,691	359,306
Posts mentioning multiple sclerosis products	168	152
Manual selection of unique posts where previous or current pregnancy was mentioned	152	70

**Table 2:** Medically relevant information shared by multiple sclerosis patients on the online posts.

Information shared in posts	Number of posts (N=70), n (%)	Illustrative text extracted from post
Gestational age	22 (31)	“I am 30 weeks pregnant”
First trimester^a^	8 (11)	“I was 6 weeks when I found I was pregnant...”
Second trimester^a^	7 (10)	“I am 27 weeks pregnant...”
Third trimester^a^	7 (10)	“I am 33 weeks pregnant...”
Time diagnosed for MS^b^	21 (30)	“I got diagnosed in 2009...”
Unplanned pregnancy	22 (31)	“...found out I was pregnant at 8 weeks and immediately stopped Gilenya...”
Planned pregnancy	8 (11)	“...stopped the medication in July to get pregnant...”
Outcome in newborns	18 (25)	“My daughter is [a] healthy one-year old...”
Previous pregnancy	10 (14)	“It’s my second baby...”
First pregnancy	7 (10)	“It’s my first pregnancy...”
Concomitant medication	5 (7)	“...Taking Methadone and Percocet as well...”
JC^c^ virus result	3 (4)	“...I am JC positive...”

^a^The first trimester (1-12 weeks), second trimester (13-28 weeks), and third trimester (29-40 weeks) according to definition available in the US Department of Health and Human Services.

^b^MS: multiple sclerosis.

^c^JC: John Cunningham.

### Content Analysis Results

Upon detailed review of the content of each post, we identified 6 main topics, which are presented in Tables 3 and 4. Patients used the pregnancy forums as an outlet for the following:

1. Describing in detail personal experiences with medicines, including changes in therapy, stopping medication, taking medication during pregnancy, and breastfeeding
2. Sharing and seeking information about MS medication in pregnancy and postpartum
3. Reporting MS progression (disease status) in this period
4. Expressing uncertainty or fears related to MS medication
5. Discussing or commenting on breastfeeding and MS medication
6. Sharing details or comments on communications with HCPs involved in the care of the pregnant mother or offspring

**Table 3:** Main topics posted by individuals on social media related to multiple sclerosis, pregnancy, and breastfeeding.

Topic		n (%)^a^
**1. Discussion about personal experiences with MS^b^** **medication in reproductive period**		56 (80)
	Switched, switching, or will change medication during pregnancy or breastfeeding	28 (40)
	Stopped, stopping, or will stop medication during pregnancy or breastfeeding	26 (37)
	Took, taking, or will take medication during pregnancy or breastfeeding	22 (31)
**2. Reporting MS disease status during and after pregnancy**		35 (50)
	Reported no relapse and healthy pregnancy	16 (22)
	Reported relapse during pregnancy	15 (21)
	Reported relapse postpartum	12 (17)
**3. Seeking and giving advice**		52 (74)
	Seeking advice about MS, pregnancy, and postpartum	36 (51)
	Giving advice about MS, pregnancy, and postpartum	16 (22)
**4. Communication with the HCP^c^**		26 (37)
	Good communication, and patient express trust in the HCP	8 (11)
	Poor communication	18 (25)
5. Discussion related to breastfeeding and MS medication		30 (42)
6. Express uncertainty and fear about MS medication in reproductive period		35 (50)

^a^Percentages are calculated using N=70 total individual posts about pregnancy and breastfeeding. The unit used was topic posted. One post may contain several pieces of information or an individual might have written about more than one pregnancy experience.

^b^MS: multiple sclerosis.

^c^HCP: health care professional.

**Table 4:** Illustrative example of posts related to each main topic and subtopic.

Topic		Illustrative text extracted from individual posts
**Sharing experiences on MS^a^ medications**		
	Stopping medication	“...I don't plan on taking anything [during] this pregnancy either.”
	Switching treatment	“...I took Copaxone throughout my pregnancy and breastfeeding and then started Tecfidera...”
	Taking medication	“...I took Copaxone throughout my pregnancy and breastfeeding under the direction of my neuro [sic]....”
**MS disease status**		
	No relapse	“...I had no issues with my MS during my pregnancy...”
	Relapse in pregnancy	“...I have very active MS had 2 relapses in 29 weeks journey. Have been on copaxone [sic] throughout and short steroids course twice...”
	Postpartum relapse	“...I didn't start flaring up until my son was over 6 months old. I've been in [sic] Tysabri since...!”
**Seeking and giving advice**		
	Seeking advice	“...I am 8 weeks pregnant and was taking my gilenya [sic] during those 8 weeks meaning the baby will be exposed to it for 2 additional months Has anyone dealt with a pregnancy like this? The doctors have such limited information.”
	Giving advice	“...MS patients are advicesd [sic] to come off their meds when trying for a baby. my understanding is that Copaxone and the interferons are perfectly ok to take until a positive pregnancy test. I'm a little bitter because I got the same advice and suffered a disabling relapse as a result. Copaxone especially is probably fine to take even during pregnancy (though now that I have finally found luck, I have chosen to stay off during pregnancy and restart after birth and yes I will be breastfeeding). Good luck.”
Breastfeeding		“...My neuro [sic] recommended a 3-day steroid infusion treatment. I had to pump and dump [sic] the whole time and for 24 hours following the last infusion...”
Express uncertainty or concerns		“...I just found out I am unexpectedly pregnant and conceived while in gilenya [sic]. Everything everyone has been telling us has made us to start thinking about terminating the pregnancy, which I really badly do not want to do. But if this child is any kind of danger I don't want to risk that. I just want someone to tell me it will be okay. I just don't know if that's realistic...”
**Communication with HCPs^b^**		
	Good communication	“...I have been on Tysabri! I talked to neuro [sic] and she completely calmed my nerves! She just had me stop all meds for now then we'll switch to Copaxone after birth...”
	Bad communication	“...My neurologist never mentioned anything, and said I can just start taking Gilenya after I give birth. She said attacks are more common after birth but didn't suggest anything to prevent them...”

^a^MS: multiple sclerosis.

^b^HCP: health care professional.

## Discussion

### Principal Findings

In this study, we performed text mining and characterization of posts acquired from pregnancy-related online forums where patients discussed MS medications. The aim of this study was to gain a better understanding of information sought by, or provided to, pregnant and breastfeeding MS patients who are active on social media. Our data show that the main topics of concern were switching, stopping, or taking medication during and after pregnancy; there was clear evidence of information seeking related to the risk of MS relapse during pregnancy or postpartum; and finally, questions were raised about breastfeeding while on medication. The most frequently observed content (approximately 80% of all relevant posts) was personal experiences with MS medications. Individuals shared their reasons for personal decisions regarding treatment; described how they felt after changes in therapy: switched, started, or stopped medication; and whether this was because of an HCP’s recommendation or because of the patient’s personal beliefs.

Patients used online forums to seek information from, and provide advice to, others (the latter occurred in 52/70, ie, 74% of posts). In 36 (51%) posts individuals asked their peers about decisions and outcomes or about experiences when taking a specific medication, queried the safety profile of certain medications, asked about the risk of MS relapses, and enquired about when to restart medical treatment postpartum. Our findings concur with the hypothesis that maternal medicine use is 1 of the 4 topics pregnant women care about most [18]. We had hoped that all of the topics would have been openly discussed with HCPs, but this was not invariably the case. In a number of posts, the patient expressed concerns that they had received medical advice from an HCP and either actively disagreed or least significantly doubted what they had been told. For example:

...I went to the infusion center for my first Tysabri treatment, the nurse said my neurologist requested a pregnancy test to rule it out before we got started. Long story short, it came back positive! My treatment was canceled. Here I am 3 years later, and pregnant with our third baby. Coincidentally, I missed my last two months of treatment (I only get it once a month) so it should be well out of my system and there shouldn't be any issues...

In comparison to our results, a Swedish study found that, when speaking with their midwives, most pregnant women (70%) did not discuss information that they had retrieved from internet despite perceiving this information to be reliable [19]. Interestingly, more than half of the study subjects searched online for topics first raised by a midwife [19]. We were not in a position to explore the reason why patients went online and searched for information about their medicines; however, a Web-based survey among women who used the internet to seek pregnancy information showed that 48.6% of respondents were not satisfied with the information provided by their respective HCPs. The majority of these respondents (46.5%) stated that they primarily turned to the internet because they did not have time during appointments to discuss their concerns [20]. Moreover, pregnant women used the internet because the information given to them by their HCPs was neither clear nor sufficient [20].

In the breastfeeding category, 30 out of 70 (42%) posts described refusal or delay in commencing MS treatment for the sake of breastfeeding, described foregoing breastfeeding to restart treatment, requested evidence of which medication might be safer to take while breastfeeding, and others commented on discarding breast milk, which was suspected to contain medication while receiving treatment (so-called *pump and dump*) [21]. Several individuals shared confusion about the risks and benefits of breastfeeding and expressed anxiety about the dilemma of caring for their own health while not doing any harm to the baby.

There is very limited information about the safety of MS medication during breastfeeding. The in vivo model for drug exposure to breast milk is suboptimal, and human milk biobanks suffer from a paucity of human breast milk samples [22]. This is paradoxical, particularly when one considers the posts concerning the *pump and dump* phenomenon. A small adjustment in behavior, based on medical advice or guidance from a midwife, could yield a range of useful samples for retention and assay within existing biobanks. In addition, it is known that pregnancy registries often have low enrollment rates [23]. In our study, just 2 of 70 posts (2%) mentioned contacting pregnancy registries. One possible solution to increase the enrollment rate of pregnancy registries and human milk biobank centers could be improving the communication to pregnant MS patients about participation both at the point of care and in online forums. A simple scripted explanation about the existence of registries, the purpose of their research, and the impact that they can have on the MS population might yield better recruitment for altruistic reasons. Encouraging individuals to participate in the available biobanks, with all exhibiting a *pump and save rather than pump and dump* philosophy after treatment, could yield valuable evidence to aid decision making.

Another important finding was the rate of unplanned pregnancies with 22 out of 70 (31%) posts describing such events and only 8 of 70 posts (11%) describing planned pregnancies. Nonetheless, in some patient information leaflets for MS medicines, both contraception and careful planning of pregnancy is clearly recommended [24]. A Danish study surveyed 590 MS patients about family planning and reported that 42% of female and 74% of male partners did not know if their MS medication was teratogenic or not. This study also reported that 10% of pregnancies during MS treatment were unplanned; 49% of these pregnancies were terminated [25].

Generally, there are gaps in current methods for collecting and analyzing data pertaining to the safety of medicines during pregnancy and lactation [26,27]. The safety of medicinal products administered during pregnancy and lactation is a complex topic that needs coordinated communication across many disciplines to obtain, analyze, and present information in a harmonized approach. Harmonized methods and metrics among different pregnancy specialties should be developed to allow better analysis of outcomes and end points [26]. In this regard, we are aware of an Innovative Medicine Initiative (IMI) project called *Continuum of Evidence from Pregnancy Exposures, Reproductive Toxicology and Breastfeeding to Improve Outcomes Now* (ConcePTION) [28]. The IMI ConcePTION project is a collaboration between public-private partners and the pharmaceutical industry to address this problem. The aim of ConcePTION is “Building an ecosystem for better monitoring and communicating safety of medicines use in pregnancy and breastfeeding: validated and regulatory endorsed workflows for fast, optimized evidence generation” [28]. Participants in this project and the authors of this paper believe that there is an important societal obligation to reduce uncertainty about the effects of medicines used during pregnancy and breastfeeding.

Furthermore, even when safety data are available, it is often not effectively communicated to patients and HCPs. On September 26, 2017, the Pharmacovigilance Risk Assessment Committee (PRAC) and the European Medicines Agency (EMA) held their first public hearing about safety concerns with the use of medications containing sodium valproate during pregnancy [29]. Patients and carers participated in the public hearing, and both mothers and affected children expressed concern about the lack of effective risk minimization communication for safety of valproate during pregnancy, despite the drug having been authorized for more than 50 years [30]. After the public hearing, the PRAC and the EMA provided new measures for comprehensive risk minimization, including the following [29]:

- A pregnancy prevention program
- Visual warning about the risk in pregnancy on the box (outer packaging)
- A patient reminder card attached to outer package for pharmacists to discuss with patients each time the medicine is dispensed
- Updated educational materials for patients and HCPs

In the valproate pregnancy prevention program, HCPs are instructed to assess patients’ potential for becoming pregnant by evaluating their individual circumstances and then assist their patients in making informed decisions. HCPs are responsible for informing their patients about the use of effective contraception methods throughout valproate treatment and to review such treatment annually. Interestingly, as an adjunct to all of these measures, a new risk acknowledgment form has been designed and implemented for patients and their HCPs to document that sufficient advice has been provided and understood [29]. Such comprehensive guidelines and the risk minimization methods adopted for valproate could serve as an example for improving and strengthening the warnings for MS medication in pregnancy.

In 2005, the EMA published guidance for assessing medicinal product risks on human reproduction and lactation [31]. In the United States, the FDA issued the Pregnancy and Lactation Labeling Rule for industry. This document provides a detailed framework for clearly communicating information to prescribers to aid improved decision making [21,32]. It is worth noting a study that reviewed medication risks during pregnancy for 172 drugs approved by the FDA between 2000 and 2010. Among these, in 97.7% of drugs, teratogenic risk in human pregnancy was *undetermined*, and the amount of data for 73.3% of these drugs was described as *none* [33]. For 468 drugs approved by the FDA between 1980 and 2000, the average time required for a drug’s risk category to be changed from *undetermined* to a more precise risk was estimated to be 27 years [33]. A Web-based survey reported that patient leaflets were not comprehensive enough to answer pregnant women’s questions and did not facilitate decision making [20]. In addition, inconsistencies have been found between the safety information concerning use during pregnancy provided in the US prescribing information and the UK summary of product characteristics for the same medicinal product [8].

### Recommendations

Evidently, there is a need to improve regulatory policy and guidance by involving not only health authorities but also HCPs, patients, and other stakeholders including the national Teratology Information Services. We suggest 2 recommendations: (1) to conduct active postmarketing surveillance and (2) to provide globally harmonized evidence-based information for the prescriber, patients, and carers in a timely manner. Inevitably, with the internet and the wide variety of social media available, information is rapidly disseminated, and patients have access to and appear to trust nontraditional sources of medical information. We anticipate that in future, it will not be permissible to take 3 decades to vary existing labelling once sufficient evidence has been generated to provide useful information to patients and prescribers.

### Conclusions

Social media can provide insight into patients’ real-life experiences with medicinal products during pregnancy as well as their struggle in comprehending the benefits and risks associated with these products. Our study showed that MS patients expressed uncertainty and concerns around reproductive health; however, social media could be utilized as a platform to engage and encourage patients to enroll in pregnancy registries and to donate samples to milk biobank research centers. The adoption of these simple methods would support the generation of essential missing safety data and would support the communication of risk minimization strategies to pregnant patients and women of childbearing potential [34].

The role of HCPs involved in supporting pregnant patients, or during early child development, should not be underestimated. HCPs could provide comprehensive information for MS patients throughout different stages of pregnancy and postpartum as well as during breastfeeding. In addition, improving safety data collection and analysis as well as implementing efficient policies in regard to practical guidelines for MS populations of childbearing age would prove advantageous. Future guidelines should address the impact of MS on pregnancy and the effect of pregnancy on MS, the risks of the occurrence of birth defects, recommendations concerning the most effective contraceptive methods, and planning pregnancy as far as possible, to allow optimal wash-out time of medication, disease control during and after pregnancy, approved medication to use in reproductive periods, and lactation guidelines following the treatment [2,35]. Further research is needed to explore the effectiveness of risk minimization methods and to improve communication between HCPs and patients to the extent that it enables and informs shared decision making.

### Limitations of the Study

Social media surveillance for medicinal product insight poses multiple challenges, which have been addressed in the literature [10,11,13]. In summary, there are technical, regulatory, privacy, and ethical considerations that need to be addressed when leveraging social media for this purpose [11]. In this study, the classifier was specifically selected to conduct research focusing on the exposure to MS medicines, not the effects of MS disease on the outcomes of pregnancy. In addition, these searches were only performed in pregnancy forums where posts related to MS medications were published. Hence, we recommend that further research be conducted in both MS and other disease-specific forums including *multiple sclerosis* term.

## Appendix

Multimedia Appendix 1 Search terms used to filter multiple sclerosis product-relevant data.

## Abbreviations

ConcePTION: Continuum of Evidence from Pregnancy Exposures, Reproductive Toxicology and Breastfeeding to Improve Outcomes Now
EMA: European Medicines Agency
FDA: US Food and Drug Administration
HCPs: health care professionals
IMI: Innovative Medicine Initiative
MS: multiple sclerosis
PII: personally identifiable information
PRAC: Pharmacovigilance Risk Assessment Committee
