# Correction: Social Media Surveillance of Multiple Sclerosis Medications Used During Pregnancy and Breastfeeding: Content Analysis

**DOI:** 10.2196/18294

**Published:** 2020-02-19

**Authors:** Bita Rezaallah, David John Lewis, Carrie Pierce, Hans-Florian Zeilhofer, Britt-Isabelle Berg

**Affiliations:** 1 Department of Clinical Research University of Basel Basel Switzerland; 2 Patient Safety Novartis Pharma AG Basel Switzerland; 3 School of Health and Human Sciences University of Hertfordshire Hatfield United Kingdom; 4 Booz Allen Hamilton Inc Boston, MA United States; 5 Department of Cranio-Maxillofacial Surgery University Hospital of Basel Basel Switzerland; 6 Hightech Research Center of Cranio-Maxillofacial Surgery University of Basel Basel Switzerland

In the article “Social Media Surveillance of Multiple Sclerosis Medications Used During Pregnancy and Breastfeeding: Content Analysis” (JMIR 2019;21(8):e13003), the author information incorrectly stated that all authors contributed equally. The correct authorship is represented by only the sequence of names, and the equal contribution footnote has been removed.

The correction will appear in the online version of the paper on the JMIR website on February 19, 2020, together with the publication of this correction notice. Because this was made after submission to PubMed, PubMed Central, and other full-text repositories, the corrected article also has been resubmitted to those repositories.

