# Supplementary material for: Social Media Surveillance of Multiple Sclerosis Medications Used During Pregnancy and Breastfeeding: Content Analysis
Source: J Med Internet Res. 2019 Aug 7;21(8):e13003. doi: 10.2196/13003 (PMC6702799; doi:10.2196/13003)
Supplement: Multimedia Appendix 1 [file jmir_v21i8e13003_app1.pdf]

Search terms used to filter multiple sclerosis product-relevant data

Product	Synonyms
alemtuzumab	alemtuzumab, Lemtrada™
teriflunomide	teriflunomide, Aubagio™
interferon beta-1a	interferon beta-1a, Avonex™, Plegridy™, Rebif™
interferon beta-1b	interferon beta-1b, Betaferon™, Betaseron™, Ferona™, Extavia™, Compesk™
glatiramer acetate	glatiramer acetate, Copaxone™, Glatopa™
daclizumab	Daclizumab
dimethyl fumarate	dimethyl fumarate, Tecfidera™
fingolimod	fingolimod, Gilenya™
natalizumab	natalizumab, Tysabri™
